# Molecular and Microscopic Challenges in Detecting *Plasmodium cynomolgi* Co-Infections with *Plasmodium vivax:* A Case Report

**DOI:** 10.3390/pathogens14070651

**Published:** 2025-06-30

**Authors:** Mohd Adilin Yaacob, Raden Shamilah Radin Hisam, Nor Parina Ismail, Noor Azian Md Yusuf, Jose Miguel Rubio Muñoz, Suhana Hashim, Tam Jenn Zhueng

**Affiliations:** 1 Parasitology Unit, Infectious Disease Research Centre, Institute for Medical Research, National Institutes of Health, Setia Alam 40170, Malaysia; raden.shamilah@moh.gov.my (R.S.R.H.); norparina@moh.gov.my (N.P.I.); noorazian.y@moh.gov.my (N.A.M.Y.); 2 Malaria & Emerging Parasitic Diseases Laboratory, Parasitology Department, National Microbiology Centre, Instituto de Salud Carlos III, Cra. Majadahonda Pozuelo Km. 2, 28220 Majadahonda, Spain; jmrubio@isciii.es; 3 Kota Bharu Public Health Laboratory (KBPHL), Kota Bharu 16010, Malaysia; drsuhanahashim@moh.gov.my; 4 Disease Control Division, Ministry of Health, Putrajaya 62590, Malaysia; tjz@moh.gov.my

**Keywords:** *P. cynomolgi*, qPCR, sequencing, microscopy, zoonotic malaria

## Abstract

The risk of non-human primate (NHP) malaria transmission to humans is increasing, with *Plasmodium knowlesi* and *Plasmodium cynomolgi* emerging as significant zoonotic threats, particularly in Malaysia. While *P. knowlesi* is well-documented, *P. cynomolgi* infections in humans remain underreported, largely due to diagnostic challenges. Routine microscopy and standard molecular diagnostic tools often misdiagnose *P. cynomolgi* infections as *P. vivax* due to morphological similarities and genetic homology. We report a new case of a human *P. cynomolgi* infection misdiagnosed as *Plasmodium vivax* in a 32-year-old male with no prior malaria history or travel to endemic countries. The initial diagnoses made by the microscopy and qPCR conducted by the Kota Bharu Public Health Laboratory in Kelantan identified the infection as *P. vivax*. However, cross-examination by the Institute for Medical Research (IMR) revealed the presence of mixed-species infection, prompting further analysis. The real-time PCR and sequencing performed at MAPELAB, Spain, confirmed the co-infection of *P. vivax* and *P. cynomolgi*. This case highlights the diagnostic limitations in detecting *P. cynomolgi*, which shares high genetic similarity with *P. vivax*, leading to potential cross-reactivity and diagnostic inaccuracies. As *P. cynomolgi* emerges as the second zoonotic malaria species after *P. knowlesi* capable of infecting humans in Southeast Asia, improved diagnostic methods are urgently needed. Enhanced molecular diagnostics and comprehensive epidemiological studies are essential to elucidate transmission dynamics, assess public health implications, and inform effective malaria control strategies.

## 1. Introduction

Malaria remains a significant public health issue worldwide, with Southeast Asian countries targeting elimination by 2030. However, the emergence of malaria parasites from non-human primates (NHPs) infecting humans presents a substantial challenge to these elimination efforts. Historically, several *Plasmodium* species that naturally infect non-human primates (NHPs) have been transmitted to humans through the bites of infected *Anopheles* mosquitoes, most notably *P. knowlesi* and *P. cynomolgi* [1]. Their ability to cross over to humans poses an escalating risk. The zoonotic transmission of *P. knowlesi* has been well-documented, and *P. cynomolgi* is emerging as an additional threat, complicating malaria control strategies in the region. For instance, in western Cambodia, epidemiological studies have uncovered 21 individuals with asymptomatic monkey malaria parasite infections, 1.9% of all identified malaria cases. Among these, 8 were infected with *P. knowlesi* and 13 with *P. cynomolgi*, signaling a growing silent reservoir of zoonotic malaria [2].

Similarly, in Thailand, an analysis of 1180 symptomatic malaria patients found that 9 (0.76%) were infected with *P. cynomolgi*, with some co-infected by *P. vivax* or *P. falciparum*, further complicating diagnosis, and treatment [3]. In Indonesia, a survey across five provinces revealed a high prevalence of *Plasmodium* infections in macaque populations, especially in Aceh. The most common species identified were *P. inui* and *P. cynomolgi*, indicating a significant potential for zoonotic transmission, particularly in areas where NHP–human interactions are frequent and mosquito vectors are present [4]. Malaysia has emerged as a critical focus in the study of zoonotic malaria, particularly due to the significant transmission of *P. knowlesi*, a parasite historically found in macaques but now recognized as a major cause of human malaria in the region. In 2023 alone, almost 2879 cases of zoonotic malaria were reported, making it the leading cause of malaria in Malaysia [5].

Research in Malaysia has focused on understanding the epidemiology, transmission dynamics, and public health impacts of *P. knowlesi* [6,7,8,9,10,11,12]. Studies have revealed that the transmission cycles involve both humans and macaques, with *Anopheles* mosquitoes acting as vectors. Research has also examined the genetic diversity of the parasite, its adaptation to human hosts, and the factors influencing zoonotic spillover. Whilst an abundance of studies have been conducted for *P. knowlesi*, another zoonotic species, *P. cynomolgi*, has not received much attention. *P. cynomolgi* may circulate undetected within human populations due to frequent misdiagnosis. These simian malaria species are often mistaken for human malaria parasites because of their similar clinical presentations and limitations in routine diagnostic methods like microscopy. As a result, the true burden of zoonotic malaria may be underestimated, with cases potentially going unnoticed.

The asexual cycle of *P. cynomolgi* is completed within 48 h, with the incubation period in the liver varying from 15 to 20 days for the B strain and 16 to 37 days for the M strain [13,14]. The first confirmed case of naturally acquired *P. cynomolgi* infection in a human was identified in Terengganu, Malaysia, in 2014 [15]. Initial microscopy suggested infection with *P. malariae* or *P. knowlesi*. However, the molecular identification conducted at the Institute for Medical Research (IMR) using a nested PCR assay developed by Snounou et al. suggested *P. vivax* infection [16]. A subsequent PCR assay combined with sequencing at MAPELAB, Spain, confirmed that the infection was due to *P. cynomolgi*.

This case highlights the ongoing challenges associated with undiagnosed or misdiagnosed *P. cynomolgi* infections, primarily due to its morphological resemblance to *P. vivax* and the limitations of commonly used PCR methods. Despite it being more than a decade since the first naturally acquired *P. cynomolgi* infection was reported in humans, diagnostic capabilities remain inadequate. The morphological similarities between *P. cynomolgi* and *P. vivax* under microscopy, coupled with the inability of standard molecular techniques to reliably distinguish between the two species, contribute to frequent misdiagnosis. In this report, we describe the diagnostic difficulties encountered when identifying *P. cynomolgi* in a mixed infection with *P. vivax* using conventional microscopic examination and molecular techniques. The findings emphasize the urgent need to develop enhanced diagnostic tools that can accurately differentiate between human and simian malaria species. Improved diagnostic accuracy is essential to better understand the epidemiology of zoonotic malaria, guide effective public health interventions, and prevent the underestimation of *P. cynomolgi* infections in endemic regions.

## 2. Case Presentation

In April 2023, a 32-year-old male presented to Jeli Health clinic, Kelantan, with a five-day history of fever. Kelantan is one of the states in Peninsular Malaysia. The patient did not report associated symptoms such as headache, chills, bleeding tendency, jaundice, vomiting, loose stools, or rash. A full blood count (FBC) was performed, with the results summarized in Table 1. A microscopic examination of a Blood Film for Malaria Parasites (BFMP) conducted at the Jeli Health Clinic indicated *P. knowlesi* infection, with a reported parasite density of 125/0 Parasites/µL. The patient was admitted, and treatment was initiated in accordance with the Ministry of Health Malaysia’s Management Guidelines of Malaria in Malaysia (2013) [17]. The case was classified as uncomplicated zoonotic malaria. Treatment for *P. knowlesi* was initiated and overseen by an Infectious Disease Physician. The patient was prescribed a standard 3-day course of Riamet^®^ (artemether–lumefantrine). A follow-up after three days of treatment confirmed parasite clearance, and the patient was discharged. Over this period of 2 years, the patient remained well with no recurrence of malaria.

## 3. Methodology

### 3.1. Isolation of DNA

DNA was extracted from whole blood using the QIAmp DNA Mini blood kit (QIAgen, Hilden, Germany) following the manufacturer’s instructions.

### 3.2. Blood Film Malaria Parasite (BFMP)

Upon the receipt of EDTA-anticoagulated blood from suspected malaria patients, thick and thin blood films were prepared in the IMR laboratory. Approximately 6 µL of blood was used for the thick smear by spreading it in a circular motion (~10 mm diameter) on a clean, grease-free slide. For the thin smear, 2 µL of blood was placed near one end of the slide and spread using a second clean slide held at a 30–45° angle to produce a feathered edge. Smears were air-dried; the thin smear was fixed with absolute methanol, while the thick smear remained unfixed. Both smears were stained with freshly prepared 3% Giemsa solution (pH 7.2) for 45 min at room temperature, rinsed gently with water, and air-dried overnight. Slides were examined under oil immersion at 100× magnification. Given the potential for low parasitemia, the entire smear area was screened before reporting it as positive or negative. Each slide was independently examined by two experienced microscopists. Parasite density was calculated using the following formula:

$$\mathrm{Parasites}/\mu L\;\mathrm{blood}=\frac{\mathrm{Number}\;\mathrm{of}\mathrm{parasites}\;\mathrm{counted}\times 8000\;\mathrm{White}\;\mathrm{cell}/\mu L}{500\;\mathrm{White}\;\mathrm{Blood}\;\mathrm{Cells}}$$

### 3.3. Nested PCR and Sequencing

A *Plasmodium*-specific nested PCR targeting the 18S ribosomal RNA gene of both human and simian malaria parasites was performed, as previously described by the IMR laboratory [16,19]. The PCR primer sequence is provided in Table S1. Briefly, the first round of nested PCR amplified the small subunit ribosomal RNA (SSU rRNA) gene using the primer pair rPLU1 and rPLU5 in a 50 µL reaction volume. The second round involved species-specific amplification using primer pairs designed for individual *Plasmodium* species, with the first-round PCR product serving as the DNA template. Capillary electrophoresis of the PCR products was performed using QiAxcel Advanced system (Qiagen, Hilden, Germany), and species identification was based on the fragment sizes of the amplicons. PCR amplicons were subsequently quantified and purified according to the instructions provided by the sequencing facility (Apical scientific, Seri Kembangan, Malaysia). The obtained nucleotide sequence was analyzed using the BLASTn program (Basic Local Alignment Search Tool) tool for species identification (https://blast.ncbi.nlm.nih.gov/), accessed on 22 May 2023.

### 3.4. abTES™ Real-Time PCR and Sequencing

abTES™ real-time PCR assays were conducted at Kota Bharu Public Health Laboratory, Kelantan, in accordance with the manufacturer’s protocol. The abTES™ reaction was performed using the abTES™ Malaria 5 qPCR II kit, which came with primer–probe mixtures and positive controls for the detection of *P. knowlesi* and four human-only *Plasmodium* species. The reaction mixture contained 5.0 µL template DNA, 6 µL reaction mix, and 2 µL of primer–probe mix, with the final volume adjusted to 25 µL with nuclease-free water. Amplification was performed by Biorad CFX96™ Real-Time PCR Detection System (Bio-Rad, Hercules, CA, USA) with the following cycling conditions: *Taq* activation at 95 °C for 2 min, followed by 45 cycles of amplification at 95 °C for 5 s and 60 °C for 20 s. The detection channels used were FAM (*P. falciparum*), HEX (*P. malariae*), ROX (*P. vivax*), Cy5 (*P. ovale*), and QUASAR 705 (*P. knowlesi*), and fluorescence was measured at the end of each cycle of amplification. The samples were considered positive by determining the threshold cycle number (Ct) at which the normalized reporter dye emission was raised above the background noise. If the fluorescent signal did not rise above the threshold at 40 cycles (Ct > 40), the sample was considered negative. Since the target gene was not specified in the assay, Whole Genome Sequencing (WGS) was employed as an alternative method. The sequencing procedure adhered to the instructions provided by the sequencing facility, and the resulting data underwent a comprehensive analysis to identify the relevant genetic information (Neoscience, Petaling Jaya, Malaysia)

### 3.5. Real-Time COI Plasmodium PCR (RT-COI 1R/5R)

The reaction mix consisted of 1× Quantimix HotSplit (Biotools, Madrid, Spain), which contained the buffer, polymerase, and dNTPs; the corresponding amounts of primers (JM-P-COI 2F and JM-P-COI 1R or JM-P-COI 5R), MALCOI 2 probe (IDT-DNA Technologies, Coralville, IA, USA), and 5 μL of template DNA in a final reaction volume of 20 μL (Table 2). The amplification conditions consisted of an initial denaturation step of five min at 95 °C, followed by 45 cycles of 10 s at 95 °C and 30 s at 60 °C, where fluorescence was read in the red channel. Amplification was performed in a Rotor-Gene Q 6 plex (QIAGEN^®^, Hilden, Germany). All samples were analyzed in duplicate, and positive controls, a known negative sample, and DNA and No-DNA isolation controls were added to each reaction to detect possible reagent contamination.

### 3.6. Nested Multiplex Malaria PCR (NM-PCR)

The NM-PCR method was able to identify the main four human malaria species in two consecutive multiplexing amplifications, including an internal reaction control in the first reaction. The reaction mixes for both reactions consisted of 1× buffer (20 mM Tris-HCl (pH 8.4), 50 mM KCl, and 15 mM MgCl) (Biotools, Madrid, Spain), 0.2 µM dNTPs, and the corresponding primers, as listed in Table 2. For the first reaction, 5 µL of DNA was used as the template in a final volume of 50 µL, and in the second amplification, 2 µL of the first reaction in a final volume of 25 µL was used. For the first reaction, the amplification conditions consisted of an initial denaturation step of five min at 95 °C, followed by 40 cycles of 20 s at 95 °C, 20 s at 58 °C, and 30 s at 72 °C and a final extension at 72 °C for 10 min. For the second reaction, the conditions involved an initial denaturation step of five min at 95 °C, followed by 35 cycles of 15 s at 95 °C, 15 s at 53 °C, and 20 s at 72 °C and a final extension at 72 °C for 10 min. Amplification was performed in a 2720 Thermal Cycler (Thermofisher Scientific, Greenville, NC, USA).

### 3.7. Nested Genus Malaria PCR (NG-PCR)

The NG-PCR method involves replacing the species-specific primers used in the second reaction of the NM-PCR method with two generic *Plasmodium* primers (Table 2) while maintaining the same reaction mix and amplification conditions.

### 3.8. Sequence Analysis and Phylogenetic Tree Construction

Amplified products were sequenced after DNA purification using the Illustra DNA and Gel Band Purification Kit (General Electric Healthcare, Dusseldorf, Germany). Sequencing was performed by cycle sequencing with the Big Dye Terminator v3.1 kit on an ABI PRISM^®^ 3700 DNA Analyzer (Thermofisher Scientific, Greenville, NC, USA). The resulting nucleotide sequences were compared using Basic Local Alignment Search Tool (BLAST)(https://blast.ncbi.nlm.nih.gov/; accessed on 19 August 2024) to identify similarities with known sequence. Subsequent multiple sequence alignment was performed using BioEdit v7.1 referencing corresponding sequences obtained from GenBank (https://www.ncbi.nlm.nih.gov/genbank/; accessed on 19 August 2024) [20]. Phylogenetic trees were constructed using TreeconW version 1.3b, employing the Neighbor-Joining method with 100 bootstrap replicates [21].

## 4. Result 

The performance of various molecular techniques and target genes in detecting *P. cynomolgi* co-infection with *P. vivax* from a single clinical sample was evaluated. While all assays consistently detected *P. vivax*, only selected methods were able to successfully identify the presence of *P. cynomolgi* (Table 3). Notably, a nested PCR assay targeting the 18S rRNA gene—employing both human- and simian-specific primer sets—demonstrated superior sensitivity and specificity, thereby reducing the risk of misdiagnosis. In contrast, approaches such as NG-PCR, RT-COIR1-PCR, and RT-COIR5-PCR followed by sequencing appeared less effective for detecting mixed-species infections, likely due to the preferential amplification of the dominant species and the potential exclusion of low-density co-infecting parasites.

## 5. Discussion

A microscopic examination of peripheral blood films—both thick and thin—remains the gold standard for malaria diagnosis and species identification. However, this method poses significant challenges due to the morphological similarities between *P. cynomolgi* and *P. vivax*, which can lead to misdiagnosis. As highlighted in this case and supported by previous studies, species misidentification is more common than previously recognized, particularly for non-human primate (NHP) malaria parasites such as *P. knowlesi* and *P. cynomolgi* [13,22,23,24]. Diagnostic accuracy is further complicated in cases of co-infection with human malaria species such as *P. vivax*, *P. falciparum*, and *P. knowlesi* [25].

To overcome these diagnostic limitations, molecular techniques offer the most reliable means of distinguishing closely related species—especially between *P. knowlesi/P. malariae* and *P. vivax/P. cynomolgi* [13,22,23,24]. Although current molecular platforms typically target the five main human *Plasmodium* spp. (including *P. knowlesi*), discrepancies between DNA sequencing and commercial methods such as that using the abTES™ Malaria 5 qPCR II Kit (AITbiotech, TIC Tech Centre, Singapore) have revealed hidden *P. cynomolgi* infections that would otherwise remain undetected. The discrepancies reflect the diagnostic complexity of zoonotic malaria and the need for robust molecular surveillance in areas where zoonotic *Plasmodium* spp. cocirculate.

**Figure 1:**
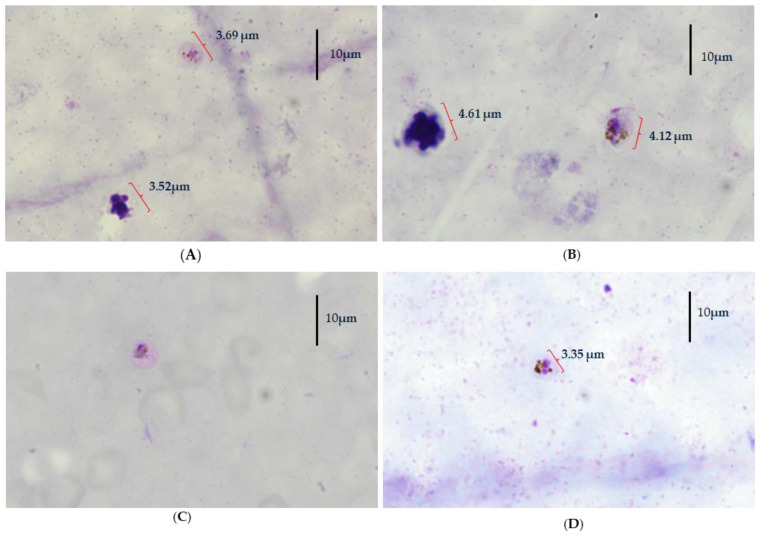
Morphological characteristics in Blood Film for Malaria Parasite (BFMP) slide that may represent *P. cynomolgi* from clinical sample. (**A**,**B**) Morphological characteristics that resemble those of *P. vivax* parasites; (**C**,**D**) morphological characteristics that may belong *to P. cynomolgi* parasite. Due to aged EDTA blood samples, morphological characteristic for parasites were determined either in thick or thin Giemsa-stained smear, wherever possible. (**A**) Thick smear—trophozoite. RBC enlarged in comparison with nucleus size of WBC. Yellowish-brown pigment scattered in cytoplasm. (**B**) Thick smear—trophozoite. RBC enlarged in comparison with nucleus size of WBC. Prominent round nucleus. yellowish-black masses of pigment. (**C**) Thin smear—trophozoite. RBC not enlarged. Large and deep red-stained chromatin. Cytoplasm was stained deep blue and lost its vacuole. Parasite shrunk and was compact, with pigment appearing in small granules, yellowish-brown and scattered in cytoplasm. Features more closely resemble those of *P. knowlesi* and *P. malariae* rather than *P. vivax*. (**D**) Thick smear—merozoite. RBC not enlarged. Large chromatin dots and yellowish-black pigment acentric to one side that resemble those of *P. knowlesi*.

Further analysis was conducted by the Institute for Medical Research (IMR). A microscopic re-examination of the blood film suggested a *P. vivax*-like morphology with a parasite density of 240/120 Parasites/µL. However, experienced microscopists observed morphological anomalies, suggesting a possible mixed infection (Figure 1). Nested PCR targeting both human and simian malaria parasites confirmed this suspicion, amplifying fragments specific to both *P. vivax* and *P. cynomolgi*. Sequencing further validated the presence of both species [6,16,19].

To corroborate these findings, the DNA sample was sent to MAPELAB, CNM-ISCIII (Madrid, Spain), for informal quality control. Using a combination of modified Nested Multiplex Malaria PCR (NM-PCR), Nested Genus-specific PCR (NG-PCR)—both targeting the SSU rRNA gene [15,26]—and *in-house* real-time PCR assays aimed at two mitochondrial COI gene regions (RT-COI R1 at 150 bp and RT-COI R5 at 350 bp), the results revealed nuanced differences. NM-PCR detected *P. vivax*, which was confirmed by sequencing. However, NG-PCR identified *P. cynomolgi* following sequencing.

**Figure 2:**
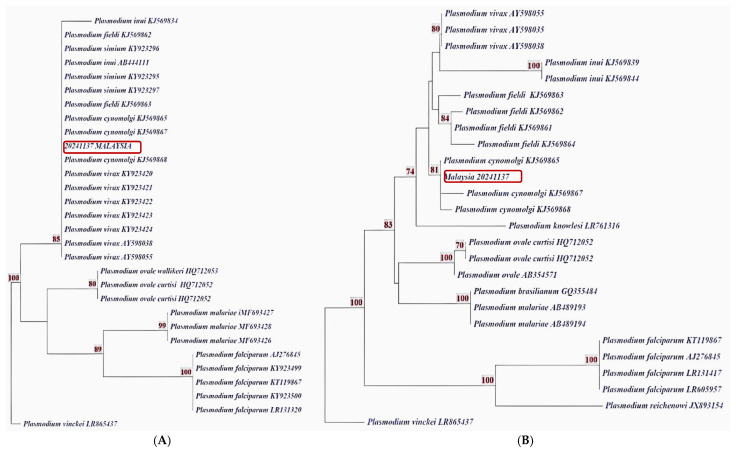
A phylogenetic tree analysis of the COI gene amplified by (A) RT-COIR1-PCR and (B) RT-COIR5-PCR for *Plasmodium* spp. The phylogenetic tree, constructed using a 150 bp COI fragment, shows the sample clustering with the *P. vivax*/*P. cynomolgi*/*P. inui* branch. This clustering suggests that the amplified sequence lacks sufficient SNP resolution to differentiate these species into distinct clades.

**Figure 3:**
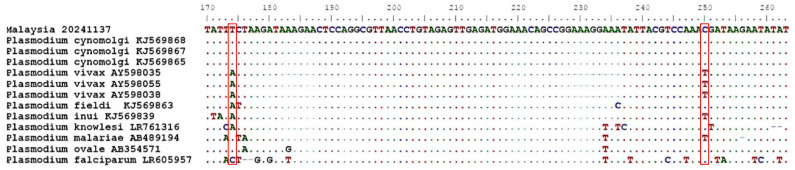
The alignment of the sequences obtained when RT-COIR5 is used. Specifically, two nucleotide substitutions were observed in the RT-COI R5 fragment: at position 174, an adenine (A) in the *P. vivax* sequence was replaced by a thymine (T) in the *P. cynomolgi* sequence, and at position 250, a thymine (T) was replaced by a cytosine (C).

The RT-COI R1 assay could not differentiate between the two species due to identical sequences in this region. In contrast, the RT-COI R5 assay detected two nucleotide substitutions (A→T at position 174 and T→C at position 250), aligning the sample with *P. cynomolgi* in the phylogenetic tree (Figure 2 and Figure 3). NM-PCR is optimized for *P. vivax* and cannot detect *P. cynomolgi*. Conversely, nested genus PCR (NG-PCR) followed by sequencing can detect *P. cynomolgi* but may fail to identify *P. vivax*, likely due to differences in parasite densities and the preferential amplification of the more abundant DNA template. A study by Lazrek et al. (2023), using artificially prepared mixed-species infections, demonstrated that the presence of a predominant species affected the cycle threshold (Ct) value, thereby complicating the detection of less abundant species [27]. This underscores the need to incorporate additional molecular targets beyond the 18S rRNA gene. In this study, the mitochondrial COI gene proved useful, with the longer 350 bp fragment offering improved resolution for haplotype analysis—effectively distinguishing *P. cynomolgi* from *P. vivax*.

This case raises several important questions for future research. Firstly, the true prevalence of *P. cynomolgi* infections in Malaysia remains unknown, suggesting the likelihood of underdiagnosed or unreported cases. Secondly, the close morphological and genetic resemblance between *P. cynomolgi* and *P. vivax* highlights the need for more sensitive and specific diagnostic tools. Finally, understanding the transmission dynamics of zoonotic malaria—including whether *P. cynomolgi*, like *P. knowlesi*, is shifting from a strictly sylvatic cycle to human-to-human transmission—is essential for shaping public health strategies and surveillance programs.

## 6. Conclusions

In cases of differential diagnosis for individuals presenting with malaria-like symptoms, particularly those with occupational or environmental exposure to forested regions, the possibility of *Plasmodium* infections, including zoonotic *P. cynomolgi* species, cannot be excluded. This report emphasized the diagnostic challenges associated with detecting naturally acquired *P. cynomolgi* infections in humans, especially in mixed infections involving *P. vivax*. Although PCR-based techniques offer high sensitivity and specificity for malaria detection, their performance in identifying *P. cynomolgi* remains inadequate.

This limitation is concerning, given the increasing recognition of simian *Plasmodium* species as potential zoonotic threats in Southeast Asia, where human–primate interactions are intensifying due to habitat encroachment and urbanization. The misdiagnosis of human and simian malaria could lead to the underestimation of the true burden of malaria, as some cases may circulate undetected in the community. To minimize the risk of misdiagnosis, it is advisable for each laboratory to develop and implement diagnostic workflows or standard operating procedures (SOPs) that are specifically tailored to the local epidemiological landscape, including the prevalence and distribution of mixed infections involving zoonotic malaria. Enhanced diagnostic capabilities will contribute to better disease surveillance, effective treatment, and the development of targeted control measures to mitigate the impact of zoonotic malaria on public health.

## Figures and Tables

**Table 1:** The full blood Count (FBC) record of the patient [18].

Parameter	Reference Range (Adult Male)	Patient Value	Remarks
Total White Blood Cell Count (×10^3^/µL)	4.00–10.00	11.30	Elevated
Hemoglobin (g/dL)	13.0–17.0	15.7	Normal
Platelet Count (×10^3^/µL)	150.0–400.0	237	Normal

**Table 2:** Oligonucleotides and probes used for molecular detection are listed, including their applications in PCR and final working concentrations. Cy5 refers to Cyanine-5, fluorescent dye detected in red channel, with excitation peak at 651 nm and emission peak at 670 nm. Second NM-PCR: size varies depending on *Plasmodium* spp.: *P. malariae* 241 bp, *P. falciparum* 370 bp, *P. ovale* 407 bp, *P. vivax* 476 bp.

Primer/Probe	Sequence (5′–3′)	PCR	Final Conc. (µM)
JM-P-COI 2F	GGTGTGTACAAGGCAACAATAC	RT-COI R1/R5	0.20
JM-P-COI 1R	CATATAACGGTAAGAAGGTTCGC	RT-COI R1	0.20
JM-P-COI 5R	CAAAGTACGCGATCTCTTGTATG	RT-COI R5	0.20
MALCOI 2	Cy5–ATTGGCACCTCCATGTCGTCTCAT–BHQ2	RT-COI R1/R5	0.15
UNR PLF HUF NewPLFshort MARshort FARshort OVRshort VIRshort NewPLFshort NewRevshort	GACGGTATCTGATCGTCTTC AGTGTGTATCCAATCGAGTTTC GAGCCGCCTGGATACCGC CTATCAGCTTTTGATGTTAG TCCAATTGCCTTCTG GTTCCCCTAGAATAGTTACA AGGAATGCAAAGARCAG AAGGACTTCCAAGCC CTATCAGCTTTTGATGTTAG CCTTAACTTTCGTTCTTG	1st NM-PCR 1st NM-PCR 1st NM-PCR 2nd NM-PCR 2nd NM-PCR 2nd NM-PCR 2nd NM-PCR 2nd NM-PCR NG-PCR NG-PCR	0.10 0.10 0.01 0.15 0.10 0.15 0.10 0.10 0.30 0.30

**Table 3:** A comparison of different approaches and target genes for identifying *P. cynomolgi* co-infections with *P. vivax* in a single clinical sample. Various methods were applied exclusively to the same patient-derived specimen. A nested PCR employing both human- and simian-specific primer sets offers significant advantages by minimizing the risk of misdiagnosis.

Target Gene	-	18S rRNA			COI	
Method	abTES™	Nested PCR	NM-PCR	NG-PCR	RT-COIR1-PCR	RT-COIR5-PCR
PCR Result	*P. vivax*	*P. vivax*, *P. cynomolgi*	*P. vivax*	*P. vivax*	Positive	Positive
Sequencing Result	*P. cynomolgi*	*P. vivax*, *P. cynomolgi*	*P. vivax*	*P. cynomolgi*	*P. vivax*-like	*P. cynomolgi*

## Data Availability

The data presented in this study are available on request from the corresponding author.

## Supplementary Materials

The following supporting information can be downloaded at: https://www.mdpi.com/article/10.3390/pathogens14070651/s1, Table S1: Sequences and annealing temperatures of species-specific PCR primers used in nested PCR.

## References

[1] Mewara A., Sreenivasan P., Khurana S. (2023). Primate Malaria of Human Importance. Trop. Parasitol..

[2] Imwong M., Madmanee W., Suwannasin K., Kunasol C., Peto T.J., Tripura R., von Seidlein L., Nguon C., Davoeung C., Day N.P.J. (2019). Asymptomatic Natural Human Infections with the Simian Malaria Parasites *Plasmodium cynomolgi* and *Plasmodium knowlesi*. J. Infect. Dis..

[3] Putaporntip C., Kuamsab N., Pattanawong U., Yanmanee S., Seethamchai S., Jongwutiwes S. (2021). *Plasmodium cynomolgi* Co-Infections among Symptomatic Malaria Patients, Thailand. Emerg. Infect. Dis..

[4] Permana D.H., Hasmiwati, Suryandari D.A., Rozi I.E., Syahrani L., Setiadi W., Irawati N., Rizaldi, Wangsamuda S., Yusuf Y. (2023). The Potential for Zoonotic Malaria Transmission in Five Areas of Indonesia Inhabited by Non-Human Primates. Parasites Vectors.

[5] Ministry of Health (MOH), Malaysia Laporan Tahunan KKM 2023(pdf). https://www.moh.gov.my/index.php/pages/view/58?mid=19.

[6] Singh B., Sung L.K., Matusop A., Radhakrishnan A., Shamsul S.G., Cox-Singh J., Thomas A., Conway D.J. (2004). A Large Focus of Naturally Acquired *Plasmodium knowlesi* Infections in Human Beings. Lancet.

[7] Vythilingam I., Tan C.H., Matusop A., Chan S.T., Lee K.S., Singh B. (2006). Natural Transmission of *Plasmodium knowlesi* to Humans by *Anopheles latens* in Sarawak, Malaysia. Trans. R. Soc. Trop. Med. Hyg..

[8] Vythilingam I., Noorazian Y.M., Huat T.C., Jiram A.I., Yusri Y.M., Azahari A.H., Norparina I., Noorrain A., Lokmanhakim S. (2008). *Plasmodium knowlesi* in Humans, Macaques and Mosquitoes in Peninsular Malaysia. Parasites Vectors.

[9] Yusuf N.M., Zulkefli J., Jiram A.I., Vythilingam I., Hisam S., Devi R., Salehhuddin A., Ali N.M., Isa M., Alias N. (2022). *Plasmodium* spp. in Macaques, *Macaca fascicularis*, in Malaysia, and Their Potential Role in Zoonotic Malaria Transmission. Parasite.

[10] Cuenca P.R., Key S., Jumail A., Surendra H., Ferguson H.M., Drakeley C.J., Fornace K. (2021). Epidemiology of the Zoonotic Malaria *Plasmodium knowlesi* in Changing Landscapes. Adv. Parasitol..

[11] Shahari S., Abdullah M.L.B., Rohimly A.A.B.I., Ashrat N.B., Amir A., Atroosh W.M.M., Fong M.Y., Lau Y.L. (2024). The Prevalence of Simian Malaria in Wild Long-Tailed Macaques Throughout Peninsular Malaysia. Sci. Rep..

[12] Hussin N., Lim Y.A.L., Goh P.P., William T., Jelip J., Mudin R.N. (2020). Updates on Malaria Incidence and Profile in Malaysia from 2013 to 2017. Malar. J..

[13] Coatney G.R., Collins W.E., Warren M., Contacos P.G. (1971). The Primate Malarias.

[14] Singh B., Daneshvar C. (2013). Human Infections and Detection of *Plasmodium knowlesi*. Clin. Microbiol. Rev..

[15] Ta T.H., Hisam S., Lanza M., Jiram A.I., Ismail N., Rubio J.M. (2014). First Case of a Naturally Acquired Human Infection with *Plasmodium cynomolgi*. Malar. J..

[16] Snounou G., Viriyakosol S., Zhu X.P., Jarra W., Pinheiro L., do Rosario V., Thaithong S., Brown K.N. (1993). High Sensitivity of Detection of Human Malaria Parasites by the Use of Nested Polymerase Chain Reaction. Mol. Biochem. Parasitol..

[17] Ministry of Health (MOH), Malaysia Management Guidelines of Malaria in Malaysia. https://www.moh.gov.my/moh/resources/Penerbitan/Garis%20Panduan/Pengurusan%20KEsihatan%20&%20kawalan%20pykit/Management_Guidelines_of_Malaria_in_Malaysia_(Final).pdf.

[18] Bhatnagar N. (2017). Dacie, and Lewis Practical Haematology (12th Edition) by B. J. Bain, I. Bates, and M. A. Laffan, Elsevier, London, 2017. Br. J. Haematol..

[19] Lee K.S., Divis P.C.S., Zakaria S.K., Matusop A., Julin R.A.M., Conway D.J., Cox-Singh J., Singh B. (2011). *Plasmodium knowlesi*: Reservoir Hosts and Tracking the Emergence in Humans and Macaques. PLoS Pathog..

[20] Hall T.A. (1999). BioEdit: A User-Friendly Biological Sequence Alignment Editor and Analysis Program for Windows 95/98/NT. Nucleic Acids Symp. Ser..

[21] Van de Peer Y., De Wachter R. (1994). TREECON for Windows: A Software Package for the Construction and Drawing of Evolutionary Trees for the Microsoft Windows Environment. Bioinformatics.

[22] Kantele A., Jokiranta T.S. (2011). Review of Cases with the Emerging Fifth Human Malaria Parasite, *Plasmodium knowlesi*. Clin. Infect. Dis..

[23] Chin W., Contacos P.G., Coatney G.R., Kimball H.R. (1965). A Naturally Acquired Quotidian-Type Malaria in Man Transferable to Monkeys. Science.

[24] Contacos P.G., Lunn J.S., Coatney G.R., Kilpatrick J.W., Jones F.E. (1963). Quartan-Type Malaria Parasite of New World Monkeys Transmissible to Man. Science.

[25] Barber B.E., William T., Grigg M.J., Yeo T.W., Anstey N.M. (2013). Limitations of Microscopy to Differentiate *Plasmodium* Species in a Region Co-Endemic for *Plasmodium falciparum*, *Plasmodium vivax* and *Plasmodium knowlesi*. Malar. J..

[26] Rubio J.M., Post R.J., van Leeuwen W.M., Henry M.C., Lindergard G., Hommel M. (2002). Alternative Polymerase Chain Reaction Method to Identify *Plasmodium* Species in Human Blood Samples: The Semi-Nested Multiplex Malaria PCR (SnM-PCR). Trans. R. Soc. Trop. Med. Hyg..

[27] Lazrek Y., Florimond C., Volney B., Discours M., Mosnier E., Houzé S., Pelleau S., Musset L. (2023). Molecular detection of human *Plasmodium* species using a multiplex real time PCR. Sci Rep.

