# Supplementary material for: Molecular and Microscopic Challenges in Detecting Plasmodium cynomolgi Co-Infections with Plasmodium vivax: A Case Report
Source: Pathogens. 2025 Jun 30;14(7):651. doi: 10.3390/pathogens14070651 (PMC12300013; doi:10.3390/pathogens14070651)
Supplement: Supplementary file 1 [file pathogens-14-00651-s001.zip › pathogens-3576513-supplementary.pdf]

Table S1: Sequences and annealing temperatures of species-specific PCR primers used in nested PCR

Plasmodium	Primers	Sequence (5' – 3')	Expected size
	RPLU1	TCAAAGATTAAGCCATGCAAGTGA	˜1,600
	RPLU5	CCTGTTGTTGTTGCCTTAAACTTC	235
	RPLU3	TTTTTATAAGGATAACTACGGAAAAGCTGT	
	RPLU4	CCCGTCATAGCCATGTTAGGCCAATACC	
Malariae	MAL1	ATAACATAGTTGTACGTTAAGAATAACCCC	144
	MAL2	AAAATTCCCATGCATAAAAAATTATACAAA	
Falciparum	FAL1	TTAAACTGGTTTGGGAAAACCAAATATATT	205
	FAL2	ACACAATGAACTCAATCATGACTACCCGTC	
Vivax	RVIV1	CGCTTCTAGCTTAATCCACATAACTGATAC	117
	RVIV2	ACTTCCAAGCCGAAGCAAAGAAAGTCCTTA	
ovale	ROVA1	ATCTCTTTTGCTATTTTTTAGTATTGGAGA	226
	RPLU2	ATCTAAGAATTTCACCTCTGACATXTG	
knowlesi	Kn1f	CTCAACACGGGAAAACTCACTAGTTTA	279
	Kn3r	GTATTATTAGGTACAAGGTAGCAGTATGC	
coatneyi	PctF1	CGCTTTTAGCTTAAATCCACATAACAGAC	
	PctR1	GAGTCCTAACCCCGAAGGGAAAGG	
cynomolgi	CY2F	GATTTGCTAAATTGCGGTCG	137
	CY4R	CGGTATGATAAGCCAGGGAAGT	
inui	PinF2	CGTATCGACTTTGTGGCATTTTTCTAC	479
	INAR3	GCAATCTAAGAGTTTTAACTCCTC	
fieldi	fieldi	GGTCTTTTTTTTGCTTCGGTAATTA	421
	PfldR2	AGGCACTGAAGGAAGCAATCTAAGAGTTTC	

assays

The genus-specific primers, rPLU1 and rPLU5, were used in the primary (nest 1) amplification followed by the species-specific primers in the nest 2 amplifications as described previously (Singh et al., 2004). The primers are based on the sequences of the small subunit ribosomal RNA genes.
